# MicroRNA-33-3p Regulates Vein Endothelial Cell Apoptosis in Selenium-Deficient Broilers by Targeting E4F1

**DOI:** 10.1155/2019/6274010

**Published:** 2019-05-22

**Authors:** Yiming Zhang, Na Wan, Tingru Pan, Xueyuan Hu, Qingqing Liu, Shu Li

**Affiliations:** College of Veterinary Medicine, Northeast Agricultural University, Harbin 150030, China

## Abstract

Selenium (Se) is a type of nutrient element. The tissues of organisms can have pathological damage, including apoptosis, due to Se deficiency. Apoptosis is an important cell process and plays a key role in vascular disease and Se-deficient symptoms. In this study, the Se-deficient broiler model was duplicated, miR-33-3p in the vein was overexpressed in response to Se-deficiency, and miR-33-3p target gene E4F transcription factor 1 (E4F1) expression was also confirmed. We utilized ectopic miR-33-3p expression to validate its function for apoptosis. The results showed that miR-33-3p-targeted E4F1 are involved in the glucose-regulated protein 78- (GRP78-) induced endoplasmic reticulum stress (ERS) apoptosis pathway. We presumed that Se deficiency might trigger apoptosis via downregulating miR-33-3p. Interestingly, the miR-33-3p inhibitor and VER-155008 (GRP78 inhibitor) partly hindered the apoptosis caused by Se deficiency. Thus, the above information provides a new avenue toward understanding the mechanism of Se deficiency and reveals a novel apoptotic injury regulation model in vascular disease.

## 1. Introduction

Selenium (Se) is an important microcrystalline nutrient in humans and in animals [1]. Se plays a significant role in many aspects of physiological function. Se can protect cardiovascular and cerebrovascular vessels [2, 3], regulate immune functions [4], decrease the incidence of cancer [5], and promote growth [6]. According to reports, Se deficiency can lead to white muscle disease in livestock [7], including muscular dystrophy [8] and exudative diathesis of chickens. Se deficiency induces vascular diseases, such as cardiomyopathy (Keshan disease) and arthropathy (Kashin-Beck disease), due to the role of Se as an essential cofactor of a number of important antioxidant enzymes [9]. Meanwhile, Se deficiency can induce tight junction injury in vein endothelial cells (VECs), increase the paracellular permeability of VECs, and arrest the cell cycle, thereby leading to, for instance, exudative diathesis and muscular dystrophy [10]. In addition, Se deficiency may induce oxidative stress in many tissues and increased oxidative stress and relative oxidative damage are mediators of vascular pathologies [11, 12]. As a vital factor in all of the stages of atherothrombosis, the Se deficiency-increased inflammatory factor expression results in many inflammatory diseases, including cardiovascular diseases [13, 14]. Broilers are susceptible to Se deficiency, especially exudative diathesis related to oxidative damage of vessels [15].

Increasing studies have indicated that low Se induces apoptosis. Apoptosis and autophagy are the two major well-known pathways of programmed cell death under stress conditions caused by Se deficiency [16]. And Se deficiency induced myocardial apoptosis in selenium-deficient rat apoptosis via the ERS pathway [17, 18]. Furthermore, lack of Se disrupts the endoplasmic reticulum in many cells and structural damage of the ER is the primary ultrastructural lesion in the pancreas of Se-deficient broilers [19]. Once the vasculature is damaged by oxidative stress, the tissues will undergo apoptosis. Researches determined that Se deficiency may destroy the vascular structure and cause blood exudation by reducing cell viability, increasing intracellular reactive oxygen species (ROS), and promoting apoptosis of vascular smooth muscle cells [20, 21]. This evidence suggests that low Se influences apoptosis in blood vessels.

miRNAs, a family of highly conserved small noncoding RNAs, have regulatory functions in apoptosis progression. miRNAs are short (22 nt), single-stranded RNAs that downregulate gene expression [22]. miRNAs can be influenced by many elements [23]. Previous researches suggested that miRNAs, such as miR-233, miR-21, and miR-31 in the human esophageal squamous cell carcinoma miRNAome, are dysregulated during zinc (Zn) deficiency. Zn is an important trace element in esophageal cancer [24]. Additionally, copper- (Cu-) mediated toxicity dysregulated miRNAs involved in neurogenesis (e.g., let-7, miR-7a, miR-128, and miR-138) via interference with the process of neurogenesis [25]. Similarly, Se can regulate the cellular miR-185 expression profile, upregulating Gpx2 and selenophosphate synthetase 2 (SEPHS2) expression [26]. And low Se might influence cancer development and progression by dysregulating miRNA expression [27]. Based on a previous study in our laboratory, we selected MiR-33-3p as a key miRNA expressed in veins. miR-33 is a highly conserved miRNA family, and the downregulation of miR-33 in mice promotes reverse cholesterol transport and regression of atherosclerosis [28]. Besides, miR-33 may attenuate neointimal hyperplasia of grafted human saphenous veins to prevent vein graft failure [29]. A previous study showed that miR-33 family members can suppress migration, invasion, and proliferation and promote apoptosis of tumor cells by regulating the expression of their target gene, abstract sirtuin 6 (SIRT6) [30]. Undoubtedly, the abnormal expressions of many miRNAs are susceptible to Se levels, including high Se and low Se.

We hypothesized that miRNAs can regulate vascular disease induced by Se deficiency. We therefore tested the role of miR-33-3p expression by analyzing its target gene (E4F1) in Se-deficient veins and VECs. Our results showed that ectopic expression of miR-33-3p regulates apoptosis caused by Se deficiency via the GRP78-mediated ERS pathway. The above information revealed that miR-33-3p is a crucial moderator of VEC apoptosis via targeting E4F1 and provides novel viewpoints for understanding mechanisms of vascular disease.

## 2. Materials and Methods

### 2.1. Reagents and Antibodies

In Table 1, the following four synthetic, chemically modified short RNA oligonucleotides were purchased from Shanghai GenePharma Co. Ltd. The Dual-Luciferase® Reporter Assay System and phRL-TK were obtained from Promega (Beijing) Biotech Co. Ltd. The pMIR-REPORT Luciferase Vector was supplied by Thermo Fisher Scientific Co. Ltd. In addition, all the nucleotides used in this work were synthetized by Sangon Biotech Company. Other chemicals were provided by Harbin Baijiesi Technology Co. Ltd.

### 2.2. Animal Tissue Samples

In this experiment, all the broilers used were approved by the Institutional Animal Care and Use Committee of Northeast Agricultural University under the approved protocol number SRM-06. Sixty male broilers (1 d old; Wei Co. Ltd., Harbin, China) were bred in an air-conditioned animal house with a normal day/night cycle. Randomly, all broilers were divided into 2 groups (30 broilers per group) and fed either a basal diet (containing 0.2 mg/kg Se) or a Se-deficient diet (containing 0.033 mg/kg Se). All other nutrients were at the standard level. The feed and tap water were supplied according to normal habits. At 45 d, when the symptoms of Se deficiency appeared in the Se-low (L) group, the broilers were euthanized with sodium pentobarbital. The veins were quickly removed and rinsed with ice-cold sterile phosphate buffer saline (PBS) (pH = 7.2). A portion of the tissue was fixed in 2.5% glutaraldehyde for ultrastructural examination, and the remaining tissue was frozen immediately in liquid nitrogen and stored at -80°C until required.

### 2.3. Ultramicroscopic Observations

For the ultrastructural examination, after fixture in 2.5% glutaraldehyde, the veins were rinsed twice for 15 min in 0.2 M phosphate buffer saline (PBS) (pH 7.2). The samples were postfixed in 1% buffered osmium tetroxide for 1 h, dehydrated through graded alcohol, and embedded in epoxy resin. Ultrathin sections were stained with uranyl acetate before examination under a transmission electron microscope (HITACHI H-7650).

### 2.4. Determination of T-AOC and ^·^OH, NO, and iNOS Levels

Total antioxidant capacity (T-AOC) and hydroxyl radical (^·^OH), nitric oxide (NO), and inducible nitric oxide synthase (iNOS) levels were determined using the manufacturer's instructions (T-AOC detection kit A015, ^·^OH detection kit A018, NO detection kit A012, and iNOS detection kit A104-1-1, respectively, Nanjing Jiancheng Bioengineering Institute). The protein concentrations of the samples were measured using the Bradford method.

### 2.5. Culture of VECs

Using pancreatin and collagenase II (Sigma-Aldrich, St. Louis, MO), broiler VECs were isolated from the tissue and cultured in Dulbecco's modified Eagle medium (DMEM) (HyClone) with 10% (*v*/*v*) fetal bovine serum (FBS) (Gibco), 100 U/mL penicillin, and 100 *μ*g/mL streptomycin. The cells were cultured in a humidified incubator containing 5% CO_2_ at 37°C.

### 2.6. Cell Transfection and Treatment

An miR-33-3p mimic, an inhibitor, and negative control oligonucleotides (Table 1) were purchased from Shanghai GenePharma Co. Ltd., which designs and chemically synthesizes the oligonucleotides. VECs were prepared for 24 h in antibiotic-free medium before transfection and culturing in six-well plates. The cells reached >70% coverage on the day of transfection. The miR-33-3p mimic, the inhibitor, or a respective negative control (NC and INC, respectively) was transfected into cells using Lipofectamine™ 2000 Reagent (Invitrogen, Carlsbad, CA, USA) in serum-free conditions for 4~6 h before changing to complete medium. The transfected cells were cultured in regular culture medium, and RNA and protein were collected 48 h after transfection. To test various parameters in subsequent studies, the cells were treated for 24 h in the presence of 30 *μ*M concentrations of VER-155008 (a specific inhibitor of GRP78, http://Selleckchem.com, Houston, TX 77014, USA) after transfection with a miR-33-3p mimic.

### 2.7. Dual-Luciferase Reporter Assay

The pMIR-REPORT plasmids for the miRNA-33-3p target E4F1 3′UTR were constructed as wild-type (WT) pMIR-E4F1 containing two tandem repeats of miRNA-33-3p response elements from E4F1 3′UTR or as mutant (MUT) pMIR-E4F1 (Table 2). The sequences of the single-stranded oligo pairs were used to generate the pMIR-E4F1 (WT and MUT). The oligonucleotides were annealed and inserted into the pMIR-REPORT vector (Thermo Fisher Scientific). The empty vector (pMIR-REPORT) was used as the negative control. VECs were seeded in 24-well plates, and cells in each well were cotransfected with pMIR-E4F1 and microRNA according to the manufacturer's protocol. Twenty-four hours after transfection, luciferase activity was measured with the Dual-Luciferase Assay System (Promega). The activity of Renilla luciferase was normalized to the activity of Firefly luciferase (Renilla LUC/Firefly LUC).

### 2.8. Quantitative RT-PCR (qRT-PCR) Analysis

Total RNA was isolated from VECs and tissues using the TRIzol extraction protocol, and the first-strand cDNA was synthesized using a qRT-PCR synthesis kit according to the manufacturer's instructions (Promega). Gene expression levels were detected via qRT-PCR with the Light Cycler® 480 System (Roche, Basel, Switzerland) using fast Universal SYBR Green Master (Roche, Basel, Switzerland). All reactions were carried out in a 10 *μ*L reaction volume. All of the primers (Table 3) were designed using Primer Premier 6.0 software (PREMIER Biosoft International, USA). The comparative 2^−△△CT^ method with *β*-actin as an endogenous control was used to calculate gene expression.

### 2.9. Western Blot (WB) Analysis

Protein extracts were subjected to SDS-polyacrylamide gel electrophoresis under reducing conditions on 10% and 12% gels and then transferred to PVDF membranes at 200 mA for 1 h in Tris-glycine buffer containing 20% methanol. Membranes were blocked with 5% skim milk for 2 h and incubated at 4°C overnight with diluted primary antibody against E4F1 (1 : 100), GRP78 (1 : 1000), activating transcription factor 6 (ATF6) (1 : 500), C/EBP homologous protein (CHOP) (1 : 500), B-cell lymphoma-2 (Bcl-2) (1:500), cysteinyl aspartate-specific proteinase 12 (Caspase12) (1 : 1000), and Caspase3 (1 : 100). Following incubation with horseradish peroxidase- (HRP-) conjugated goat anti-rabbit IgG (1 : 5000, Santa Cruz Biotechnology, USA), the bands were detected using an ECL reaction. The optical density (OD) was determined using an ImageJ VCD gel imaging system.

### 2.10. ROS Detection Assays

The levels of ROS were measured using a detection kit from the Beyotime Institute of Biotechnology (Nantong, China) according to the manufacturer's instructions. Next, fluorescence distribution of the VECs was detected by fluorospectrophotometer analysis at an excitation wavelength of 488 nm and an emission wavelength of 525 nm.

### 2.11. Fluorescence Microscopy of VECs

The control group, mimic group, inhibitor group, and corresponding negative controls were seeded into 6-well plates (1 × 10^6^ cells/well). Then, 200 *μ*L of Hoechst 33258 dye was added to the culture and the cells were incubated for 30 min. The cells were washed three times with PBS, and antifade mounting medium was added to the cells.

### 2.12. TUNEL Assay

To analyze Se deficiency-induced apoptosis, a TUNEL assay was performed to detect DNA fragmentation with a commercial cell apoptosis detection kit (Roche, USA) according to the manufacturer's protocol. The apoptotic cells were photographed with an inverted microscope (DMI4000, Leica, Germany) at 200x magnification.

### 2.13. Detection of Endothelial Nitric Oxide Synthase (eNOS) Levels In Vivo and In Vitro

An eNOS ELISA Kit (Nanjing Jiancheng Bioengineering Institute) was used for measuring eNOS concentration of vein tissues in Se-deficient broilers and cultured VECs. The OD of each hole is measured in order with the blank hole as zero, and the 450 nm wavelength is measured in order and calculated using ELISAcalc.

### 2.14. Statistical Analysis

All experiments were performed in triplicate. The data analysis was performed using SPSS statistical software for Windows (version 23; SPSS Inc., Chicago, IL, USA). Differences between different groups were assessed using Student's *t*-test or one-way ANOVA. The data are expressed as the means ± standard deviation. Differences were considered to be significant at *P* < 0.05.

## 3. Results

### 3.1. Se Deficiency Induces VEC Apoptosis

**Figure 1:**
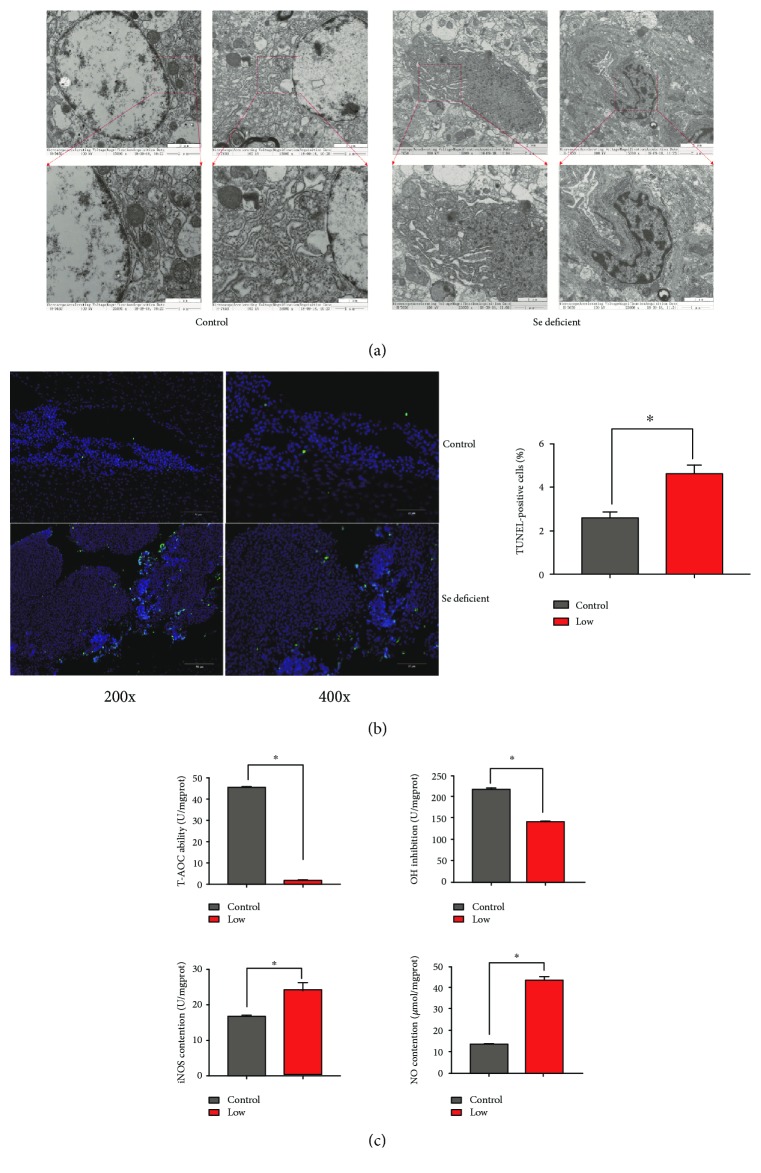
The effects of Se-deficient diet on broiler vein tissues. (a) Ultramicroscopic observations (15000x or 25000x) of broiler vein tissues in the control group and Se-deficient group. (b) Apoptosis was measured by using TUNEL assay (200x or 400x) in the vein tissues of broilers from the control group and Se-deficient group. (c) The effects of Se deficiency on the ability of T-AOC, inhibition of ^·^OH, and contention of NO and iNOS in broiler veins are showed. ∗ shows significant difference (*P* < 0.05).

To determine the effect of Se deficiency on broiler veins, we observed changes in the veins of Se-deficient broilers via transmission electron microscopy. In Figure 1(a), we chose several typical pictures to illustrate Se deficiency-induced transformations in the intercellular structure of VECs. The VECs possessed complete cell membranes and normal cytoplasmic organelles in the control group. However, the VECs in the Se-deficient group did not have a plasmosome. The VECs had shrunken nuclei, broken nuclear membranes, and chromatin concentration and margination. Moreover, some vacuoles of different sizes and quantities were existent in the endochylema. In addition, the endoplasmic reticulum was broken, swollen, and showed partial ribosomal detachment.

We additionally carried out a TUNEL assay to understand the effect of Se deficiency on apoptosis in broiler veins. The results are shown in Figure 1(b). A low-Se diet remarkably increased the number of TUNEL-positive nuclei compared to the control group. Quantification of the apoptotic index (Figure 1(b)) showed that the number of TUNEL-positive cells increased significantly in the Se-deficient group compared with the control group (*P* < 0.05).

To confirm the influence of Se deficiency on oxidative stress in broiler veins, we determined T-AOC and inhibition of ^·^OH and the concentrations of NO and iNOS in broiler veins (Figure 1(c)). The results showed that, compared to the control group, the T-AOC and inhibition of ^·^OH decreased remarkably in the Se-deficient group (*P* < 0.05) and the concentration of NO and iNOS increased significantly in the Se-deficient group (*P* < 0.05). Accordingly, the oxidation resistance of VECs in broiler veins was weakened and oxidative stress occurred. The results revealed that Se deficiency induced broiler vein apoptosis.

### 3.2. VEC Culture In Vitro

**Figure 2:**
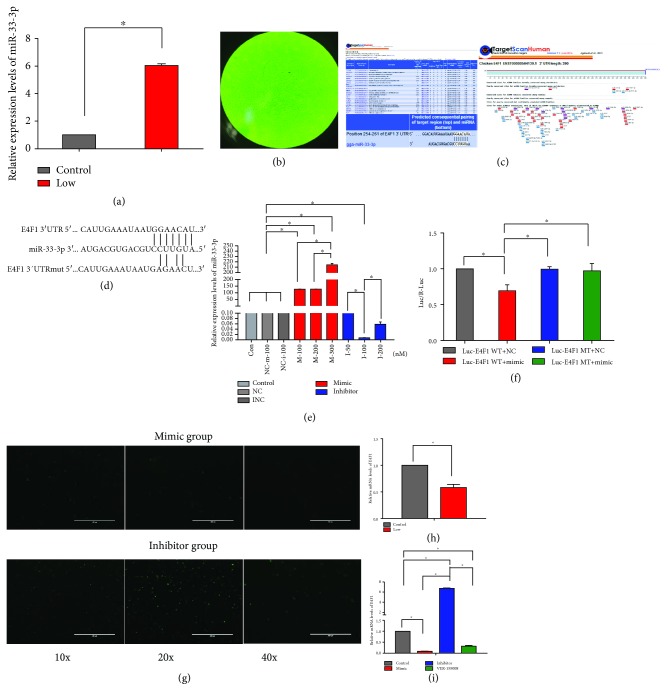
Different miRNA and mRNA expression profiles and qRT-PCR detection and luciferase activity assay. (a) Relative expression levels of miR-33-3p in tissues of the control group and Low-Se group via qRT-PCR. (b) Configuration of the first generation of VECs without treatment. (c) E4F1, a target of miR-33-3p, was predicted by TargetScan software. (d) Wild-type (WT) and mutant-type (MT) (scrambled oligonucleotide) putative miR-33-3p target sequences of E4F1 3′TR. (e) To determine the most appropriate transfection concentration in VECs, we transfected different concentrations of miR-33-3p mimic, miR-33-3p inhibitor, the mimic negative control, and the inhibitor negative control for 24 h. miRNA expression of miR-33-3p was detected via qRT-PCR. The optimum concentrations of miR-33-3p mimic and miR-33-3p inhibitor used in this paper were always 300 nM and 100 nM, respectively. (f) Expression of luciferase with the putative miR-33-3p target sites in WT or MT from E4F1 was detected in a luminometer. (g) To prove that transfection is done, efficiency is showed. (h) E4F1 mRNA expression levels in tissues were measured by qRT-PCR. (i) E4F1 mRNA expression levels in VECs were measured by Western blot and ImageJ. ∗ shows significant difference (*P* < 0.05). The results were expressed as the mean ± SD of triplicate cell cultures.

To study the role of miR-33-3p in VECs, we cultured VECs for the following experiment. Using a microscope, we observed that a mass of first-generation VECs grouped together, while a portion of them were scattered. After approximately 24 h of incubation, VECs grew rapidly; approximately 2 d later, cells entered a stable period and reached 80% coverage at 4 d. In the current state, the cells showed a typical slab stone-like arrangement and a clear nucleus (Figure 2(b)).

### 3.3. miR-33-3p Is Involved in Se Deficiency-Induced VECs in the Broiler by Targeting E4F1

According to the previous high-throughput sequencing results of the laboratory, miR-33-3p may be the specific miRNA involved in Se deficiency in veins. We detected the Se-specific miRNA miR-33-3p, which is upregulated in the Se-deficient group compared with the control group. To confirm this result, quantitative reverse transcription-polymerase chain reaction (qRT-PCR) was used to verify the miR-33-3p levels in vivo. Consistent with the predicted results, miR-33-3p was elevated 6 times higher in the Se-deficient group compared with the control group (Figure 2(a)).

To discover how miR-33-3p elicits its effect on VEC apoptosis triggered by Se deficiency, we selected downstream target genes of miR-33-3p. We determined that E4F1 was a potential target of miR-33-3p using TargetScan software (Figure 2(c)). Due to the critical role of E4F1 in apoptosis, we further tested whether E4F1 was a target protein for miR-33-3p. We detected target genes through qRT-PCR in vivo and in vitro (Figures 2(h) and 2(i)). The relative mRNA level of E4F1 was reduced by 50% in venous tissue in the Se-deficient group compared with the control group. In addition, the expression of E4F1 was decreased in response to decreasing the level of miR-33-3p and increased in response to elevating the level of miR-33-3p in VECs that were transfected with an miR-33-3p mimic and an miR-33-3p inhibitor. To evaluate optimal transfection concentrations of miR-33-3p, we transfected the mimic and inhibitor into cultured VECs and evaluated the expression of miR-33-3p by qRT-PCR. Compared with the control group, miR-33-3p levels were significantly increased by 214-fold in the mimic group, whereas levels were decreased by 138-fold in the inhibitor group (Figure 2(e)). The transfection efficiency is shown in Figure 2(g). Thus, the miR-33-3p differential expression model in vitro was effective and could be used for subsequent experiments. To determine the targeting function of miR-33-3p, we constructed vector plasmids containing wild-type or mutant 3′UTR of E4F1 fused to the luciferase gene. VECs transfected with a miR-33-3p mimic decreased the luciferase reporter activity from the 3′UTR of wild-type E4F1 but not of that containing the 3′UTR of mutant E4F1 (Figures 2(d) and 2(f)). These results suggest that E4F1 is a specific downstream target gene of miR-33-3p and may be involved in VEC apoptosis triggered by Se deficiency.

### 3.4. miR-33-3p with Mediation of the ERS Pathway and Apoptosis Pathway in VECs

**Figure 3:**
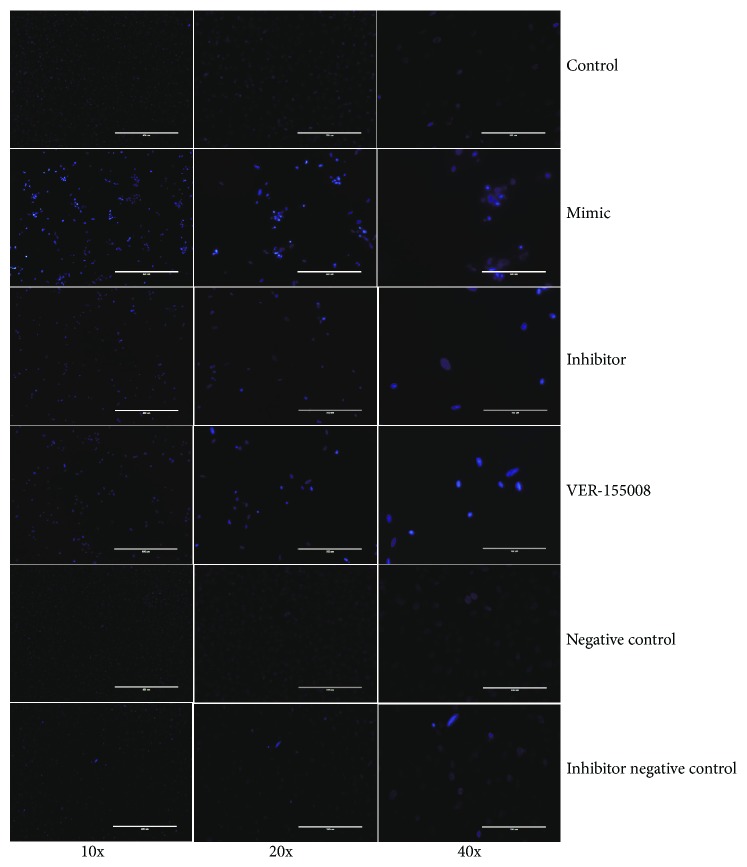
miR-33-3p-mediated modulation of E4F1-mediated apoptosis. Detection results of staining with the Hoechst 33258 of VEC-transfected mimic, inhibitor, VER-155008, negative control, inhibitor negative control, and control VECs. The normal cells are stained nattier blue. And the apoptosis cells are stained bright blue.

To understand the pathophysiological role of miR-33-3p and its target gene, E4F1, in the vein, we determined whether miR-33-3p was involved in apoptosis in VECs. Fluorescence microscopy was used to estimate VEC apoptosis caused by the regulation of miR-33-3p (Figure 3). There was a significant increase in the number of bright-blue cells in the miR-33-3p mimic group compared to the control group. The opposite result was seen in the VECs with a knockdown of miR-33-3p. Compared to the mimic group, VEC apoptosis in the VER-155008 group showed a downward trend.

**Figure 4:**
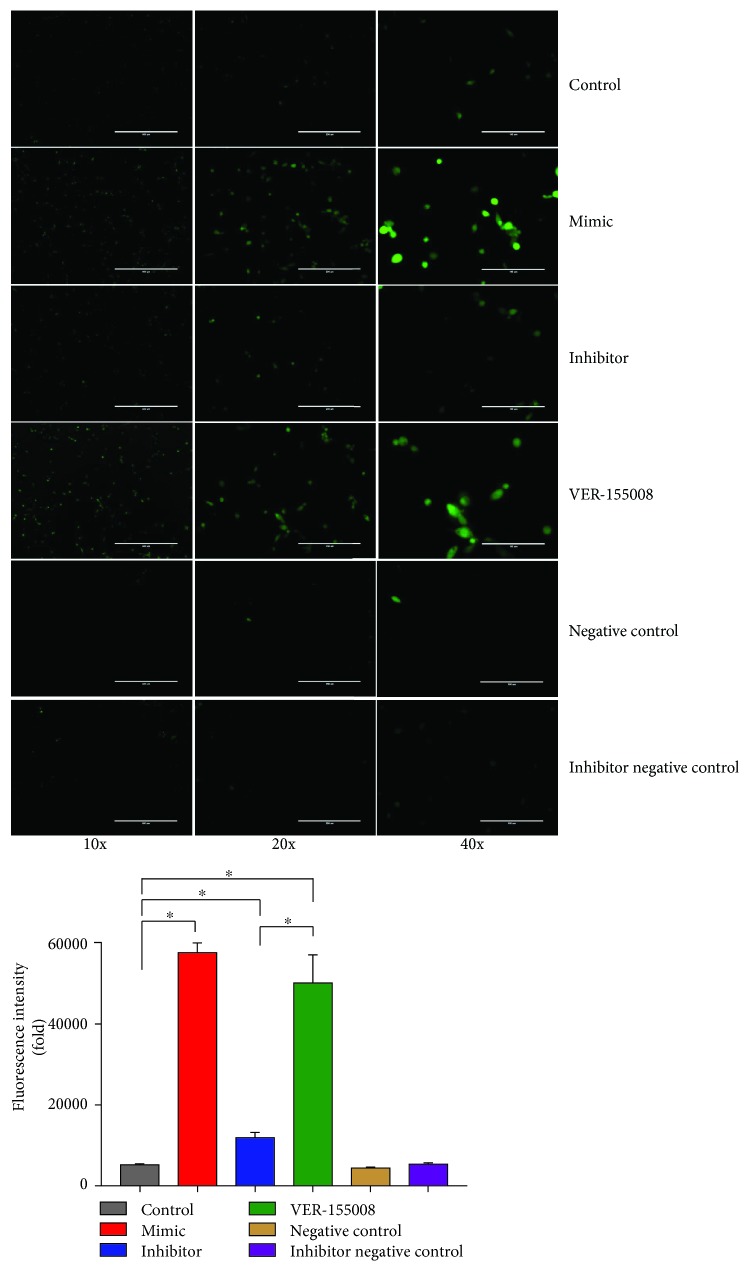
miR-33-3p-mediated modulation of redox states. ROS generation was performed by immunofluorescence using DCFH-DA (green fluorescence, 5 mM) in VECs. VECs were transfected with mimic, inhibitor, and VER-155008. And VECs were visualized using fluorescence microscopy. ∗ shows significant difference (*P* < 0.05). The results were expressed as the mean ± SD of triplicate cell cultures.

To explore whether upregulation of miRNA can induce oxidative stress in cultured VECs, we tested the levels of ROS (Figure 4). Downregulation of E4F1 caused by the miR-33-3p mimic-treated VECs significantly increased the level of ROS by 11-fold (*P* < 0.05). In contrast, in the miR-33-3p inhibitor group, the levels of ROS were not obviously changed compared to the miR-33-3p NC and INC groups. In addition, we observed that ROS levels in the VER-155008 group also increased 9-fold compared with the control group (*P* < 0.05). In other words, VER-155008 inhibits the expression of GRP78. Oxidative stress caused by E4F1 downregulation was unaffected. The above data suggested that the decrease in E4F1 caused by miR-33-3p upregulation induced oxidative stress in VEC in broilers and then influenced subsequent physiological reactions.

**Figure 5:**
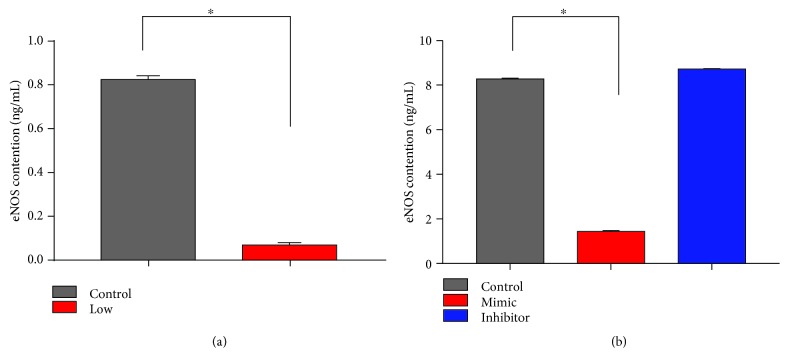
The levels of eNOS in vivo and in vitro. (a) The eNOS ELISA Kit was used to detect the expression levels of eNOS in the control group and the Se-deficient group of venous tissues. (b) The concentration of eNOS in the control group, miR-33-3p mimic group, and miR-33-3p inhibitor group of broiler VECs. ∗ shows significant difference (*P* < 0.05). The results were expressed as the mean ± SD of triplicate cell cultures.

Owing to eNOS is one of the most important indicators of vascular disease. We detected the level of eNOS in vivo and in vitro by the eNOS ELISA Kit (Figure 5). In vivo (Figure 5(a)), the level of eNOS in the control group is higher compared with that in the low-Se group (*P* < 0.05). And in vitro (Figure 5(b)), the eNOS concentration in the mimic group was lower than that in the control group (*P* < 0.05). In contrast, the eNOS concentration in the inhibitor group is increasing a bit compared with that in the mimic group (*P* < 0.05). The above data suggested that oxidative stress, as a cause of eNOS dysfunction, indeed appeared in the injury of the blood vessel induced by Se deficiency.

**Figure 6:**
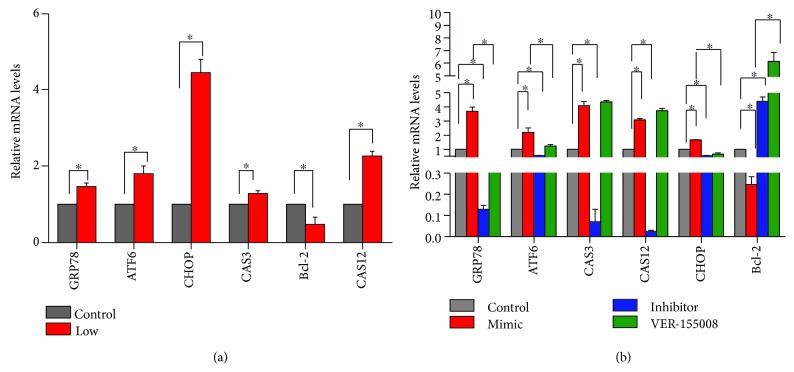
Different mRNA expression levels of ERS pathway-related and apoptosis-related genes in vivo and in vitro. (a) The mRNA expression levels of GRP78, ATF6, CHOP, Caspase3, Caspase12, and Bcl-2 in the control group and the Se-deficient group of venous tissue. (b) The mRNA expression levels of GRP78, ATF6, CHOP, Caspase3, Caspase12, and Bcl-2 in the control group, miR-33-3p mimic group, miR-33-3p inhibitor group, and VER-155008 group, respectively, of broiler VECs. ∗ shows significant difference (*P* < 0.05). The results were expressed as the mean ± SD of triplicate cell cultures.

**Figure 7:**
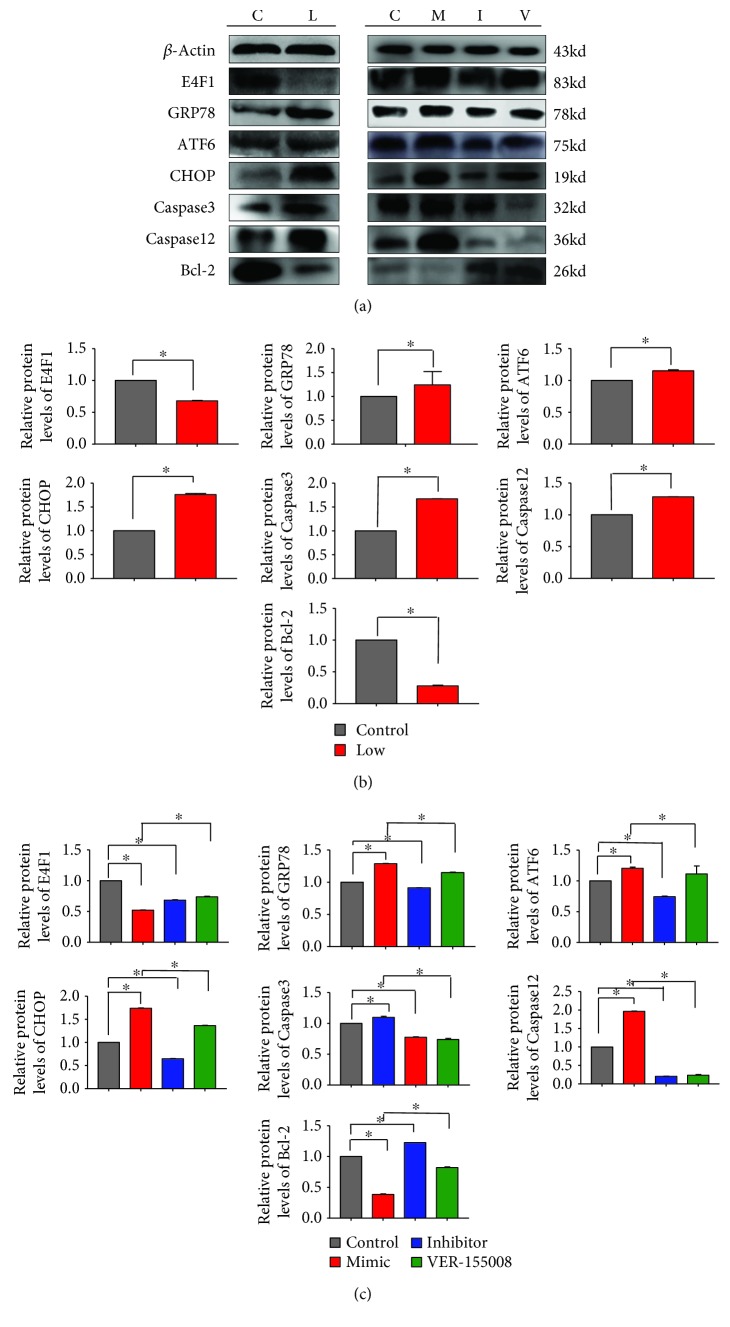
Different protein expression levels of ERS pathway-related and apoptosis-related genes in vivo and in vitro. The protein levels of E4F1, GRP78, ATF6, CHOP, Caspase3, Caspase12, and Bcl-2 in VECs transfected with miR-33-3p mimic and miR-33-3p inhibitor and those treated with VER-155008 were detected in vivo and in vitro by Western blot and ImageJ. ∗ shows significant difference (*P* < 0.05). The results were expressed as the mean ± SD of triplicate cell cultures.

Additionally, in order to study the expression levels of relative genes in VECs transfected with the miR-33-3p mimic and miR-33-3p inhibitor, we evaluated the expression of ERS-related genes and apoptotic genes by qRT-PCR and WB in primary culture VECs. The miR-33-3p increased the release of GRP78, ATF6, CHOP, Caspase12, and Caspase3 and decreased the release of E4F1 and Bcl-2, and the same results were shown at the protein level. Interestingly, the opposite results were generated in the knockdown-miR-33-3p group. We also observed that the expression of E4F1 was not significantly changed following VER-155008 treatment (Figures 6(b) and 7). The above results suggested that miR-33-3p regulates E4F1-mediated ERS-related genes and apoptosis-related genes.

According to the previous VEC experiments, we detected gene expression in broiler vein tissue further. Using qRT-PCR and WB, we evaluated the expression levels of ERS-related genes and apoptotic-related genes. Se deficiency elevated the levels of GRP78, ATF6, CHOP, Caspase12, and Caspase3 and reduced the levels of E4F1 and Bcl-2 (Figures 6(a) and 7). The above results were consistent with the data from the transfection of miR-33-3p in VECs and suggested that Se deficiency could modulate the expression of the ERS-related genes and apoptosis-related genes, thereby causing VEC apoptosis.

## 4. Discussion

Se can exert many biological protection functions to maintain the normal homeostasis of the body. In addition, decreased Se concentrations can induce apoptosis in a variety of tissues. Relative reports suggested that Se deficiency promoted liver apoptosis induced by the oxidative-endoplasmic reticulum stress pathway in the broiler [31]. And apoptosis can be induced by dietary Se deficiency at different time points in the thigh, pectoral, and wing muscles of chickens [32]. Based on previous reports, we observed several apoptotic characteristics in venous tissues of Se-deficient broilers. In addition, miR-33, a microRNA family involved in various signaling pathways, could regulate apoptosis by changing its expression due to extraneous factors [33]. Meanwhile, E4F1 might influence apoptosis through the oxidative stress pathway. Therefore, in this study, we discussed the regulation of apoptosis via oxidative stress pathways induced by miR-33-3p targeting E4F1 in Se-deficient broilers.

Recently, research concerning miRNAs has focused on modulations of developmental and physiological processes. miRNAs may cause pathological phenomena induced by low Se. Se deficiency induced myocardial necrosis, cardiac dysfunction, and splenic apoptosis by regulating miR-200a-5p, miRNA-155, and other miRNA levels [34–36]. In addition, miRNAs may regulate physiological mechanisms, such as protecting hepatocytes and adjusting autophagy through the ERS pathway [37, 38]. As an important multifunctional miRNA family, miR-33 is involved in many mechanisms and signaling pathways. miR-33 can regulate cell proliferation, cell cycle progression, glucose metabolism, hematopoietic stem cell self-renewal, autophagy, and host lipid metabolism. In addition, miR-33 can also regulate biliary transporter expression and mediate the mechanism of statin- and diet-induced hepatotoxicity [39–42]. But the role of miR-33-3p in Se deficiency-induced apoptosis is not clear. In this study, we discovered that the expression of miR-33-3p was upregulated in Se-deficient veins while E4F1 was downregulated. The interplay between miR-33-3p and E4F1 affected the process of apoptosis.

E4F1 was involved in key signaling pathways that are commonly dysregulated during cell transformation [43]. Moreover, relative studies indicate that E4F1 deficiency results in increasing levels of intracellular ROS, leading to oxidative stress [33, 44, 45]. And E4F1 may contribute to the viability and proliferation of HBV-infected cells [46]. In addition, E4F1 also decreases expression of the cyclin A promoter and downregulates cell cycle progression. And according to previous research, E4F1 stabilizes the tumor suppressor protein p53 and activates it to induce growth suppression via exerting an unconventional ubiquitin-ligase activity [47]. We first predicted that miR-33-3p binds to the 3′UTR of E4F1 by using TargetScan software and detected the luciferase activity of E4F1 3′UTR in the wild type or mutant. miR-33-3p was inhibited by the E4F1 3′UTR. Then, we confirmed that overexpression or knockdown of miR-33-3p could down- or upregulate the level of E4F1 in the cultured VECs. Therefore, we affirmed that E4F1 was a target gene belonging to miR-33-3p. In addition, the change of E4F1 expression may affect ROS levels. Hatchi et al. and Wang et al. showed that depletion of E4F1 can induce ROS-mediated death in myeloid leukemia cell lines by increasing the expression of ROS, causing oxidative stress [45, 48]. The oxidative stress aggravates apoptosis. Apoptosis, the regulated destruction of a cell, is a complicated process. Se deficiency decreases the activity of antioxidant enzymes, resulting in metabolic disturbances and aggravating oxidative damage [49]. The internal environment of the endoplasmic reticulum can be disturbed during oxidative stress. Oxidative stress induces constant ERS [31]. As the ROS content increases to a certain level, ROS can cause indiscriminate damage to biological molecules, leading to loss of function and even cell death [50]. Oxidative stress occurs when the level of ROS overwhelms the intrinsic antioxidant defenses. In this study, to understand antioxidant defenses, we estimated the level of ROS in VECs. In addition, NOS is an isozyme found in endothelial cells, macrophages, neurophagocytes, and nerve cells. And NOS includes multiple subtypes, such as iNOS and eNOS. Meanwhile, previous researches suggested that concentrations of iNOS and eNOS both can be detected in vascular disease, including oxidative stress. And nitric oxide derived from iNOS has toxic effect in general, but that derived from eNOS has protective effect. But a relative study considered that under chronic proinflammatory conditions, the expression of the inducible NOS is seen in endothelia and other cell types usually [51]. The expression of iNOS was considered as an early marker for inflammatory processes [52]. High-output NO derived from synthesis is thought to contribute to tissue destruction in a number of chronic inflammatory diseases [53]. In addition, relative studies considered that the effect of suppressed cellular apoptosis by kallistatin was blocked by the knockdown of eNOS expression [54]. The detection of iNOS and eNOS levels contributes to the study on the oxidative stress process. The results showed oxidative stress. Oxidative stress can activate many cellular processes, including ERS. Soliman et al. observed that apoptosis increased if the ERS mechanism was critically overloaded [55].

GRP78 is an important protein in ERS [56]. Researches stated that GRP78 was involved in unfolding protein reactions and protection mechanisms during ERS and the upregulation of GRP78 was an indication of ERS [57]. GRP78 combines with transmembrane proteins in the endoplasmic reticulum without ERS when GRP78 is inactive. However, GRP78 can combine with unfolded protein after dissociating from transmembrane proteins. The rest of the transmembrane proteins activate unfolded protein reactions, which promotes recovery of the endoplasmic reticulum [56, 58]. However, this assay can precipitate apoptosis with serious ERS, and it promotes apoptosis induced by activated downstream apoptotic signaling molecules [59–61]. According to the results of the experiment, GRP78 promoted the expression of ATF6 and CHOP. ATF6 is part of the basic leucine zipper family of transcription factors and is involved in the ERS pathway. It can activate expression of GRP78 and other genes induced by ERS [62]. Overexpression of CHOP results in ERS-induced apoptosis [63–65]. In addition, ER-resident Caspase12, an ER-specific apoptosis pathway signaling molecule, is activated, leading to Caspase3 activation and cellular death [66]. Meanwhile, the decreased expression of Bcl-2 increases apoptosis and vice versa [67, 68]. Furthermore, VER-155008 (a specific inhibitor of GRP78) significantly inhibits GRP78 expression, decreases mRNA levels of CHOP, relieves the dilatation of the endoplasmic reticulum (ER), and alleviates apoptosis [69, 70]. Therefore, in this study, we studied whether VER-155008 restrains apoptosis or contributes to apoptosis.

## 5. Conclusions

This study showed that Se deficiency could enhance the expression level of miR-33-3p. Meanwhile, we confirmed that E4F1 is the target gene of miR-33-3p. Subsequently, we found that overexpressing miR-33-3p regulated oxidative stress and ERS induced by the suppression of E4F1, ultimately leading to apoptosis in the VECs of broilers. miRNA regulation may be a novel mechanism of low Se-induced vascular diseases. miRNA regulation can provide new insights into the potential mechanisms behind low Se-induced vascular diseases.

## Figures and Tables

**Table 1:** miR-33-3p mimic, inhibitor, and negative control sequence.

Name	Sequence (5′~3′)
Mimic (M)	AAUGUUCCUGCAGUGCAGUA CUGCACUGCAGGAACAUUUU
Negative control (NC)	UUCUCCGAACGUGUCACGUTT ACGUGACACGUUCGGAGAATT
Inhibitor (I)	UACUGCACUGCAGGAACAUU
Inhibitor negative control (INC)	CAGUACUUUUGUGUAGUACAA

**Table 2:** The single-stranded oligo pairs used to generate the pMIR-E4F1 (WT and MUT).

Primer	Sequence (5′~3′)
E4F1-1	ACGATGGTGGCATCTGAGGACATTGAAATAAT**GGAACAT**ACAGGAGAGTTTGTTATTGCCTCCCAGGAAGGG
E4F1-2	CCCTTCCTGGGAGGCAATAACAAACTCTCCTGT**ATGTTCC**ATTATTATTATTTCAATGTCCTCAGATGCCACCATCGT
E4F1-MUT-1	ACGATGGTGGCATCTGAGGACATTGAAATAAT**GAGAACT**ACAGGAGAGTTTGTTATTGCCTCCCAGGAAGGG
E4F1-MUT-2	CCCTTCCTGGGAGGCAATAACAAACTCTCCTGT**AGTTCTC**ATTATTATTATTTCAATGTCCTCAGATGCCACCATCGT

**Table 3:** The primers used in the present study.

Gene	Forward primer (5′~3′)	Reverse primer (5′~3′)
miR-33-3p	AATGTTCCTGCAGTGCAGTA	miRcute plus miRNA qRT-PCR detection kit reverse primer
U6	CACGCAAATTCGTGAAGCGTTCCA	miRcute plus miRNA qRT-PCR detection kit reverse primer
E4F1	GCAGGTCGCTATGACACTGG	AATGTCCTCAGATGCCACCA
GRP78	TCGGCTAACACCAGAGGAGA	ACGCATAGCTCTCCAGCTCA
ATF6	GTGGTACAGCTGCAGGCTTC	GCTGAGCCATTTCCAGCAGT
CHOP	AGTGTGCTGTGAGCTGGATG	CTTCCGCTTTGTCCTCTGCC
Bcl-2	GATCGTCGCCTTCTTCGAGT	CAGGTGCCGGTTCAGGTACT
Caspase12	TCAACAACCGTAACTGCCAGA	GCAAGAGCCGACCATGAGTA
Caspase3	CTGAAGGAACACGCCAGGA	CTGTCGAGTGGAGCAGGATTC

## Data Availability

The data used to support the findings of this study are included within the article.

## Abbreviations

Se: Selenium
miRNA: MicroRNA
VECs: Vein endothelial cells
ERS: Endoplasmic reticulum stress
UPR: Unfold protein reaction
E4F1: E4F transcription factor 1
GRP78: Glucose-regulated protein 78
ATF6: Activating transcription factor 6
CHOP: C/EBP homologous protein
Caspase: Cysteinyl aspartate-specific proteinase
Bcl-2: B-Cell lymphoma-2
ROS: Reactive oxygen species
UTR: Untranslated region
qRT-PCR: Quantitative real-time PCR
T-AOC: Total antioxidant capacity
^·^OH: Hydroxyl radical
NO: Nitric oxide
iNOS: Inducible nitric oxide synthase
OD: Optical density
Gpx: Glutathione peroxidase
Txnrd1: Thioredoxin reductase 1
Zn: Zinc
Cu: Copper
SEPHS 2: Selenophosphate synthetase 2
SIRT 6: Sirtuin 6
PBS: Phosphate buffer saline
DMEM: Dulbecco's modified Eagle medium
FBS: Fetal bovine serum.
